# Supplementary material for: Applicability of Diagnostic Criteria and High Prevalence of Familial Dysbetalipoproteinemia in Russia: A Pilot Study
Source: Int J Mol Sci. 2023 Aug 24;24(17):13159. doi: 10.3390/ijms241713159 (PMC10487848; doi:10.3390/ijms241713159)
Supplement: Supplementary file 1 [file ijms-24-13159-s001.zip › Supplementary Materials_S1_revised.pdf]

Supplementary Table S1. The clinical interpretation of the detected *APOE* variants, associated with the autosomal dominant FD.

Number of subjects, total (ESSE-Ivanovo)	Variant	Genomic coordinates (GRCh37)	Reference allele	Alternative allele	Variant class	Consequence	HGVSc (NM_000041.4)	HGVSp (NP_000032.1)	AF, gnomAD [1], %	ACMG/ AMP2015	Clinical Significance
5 (3)	rs121918393 *APOE2* Heidelberg	chr19:45412013	C	T	SNV	missense	c.460C>T	p.Arg154Cys	0.008979	PM1 [1], PM2, PP1_Moderate [1], PP3 (REVEL=0.898), PP4, PP5	likely pathogenic
4 (1)	rs267606664	chr19:45411987	G	A	SNV	missense	c.434G>A	p.Gly145Asp	0.01532	BS1, PP4, PP1, REVEL = 0.581	variant of uncertain significance
6 (3)	rs199768005	chr19:45412314	T	A	SNV	missense	c.761T>A	p.Val254Glu	0.04515	BS1, PP4[2], REVEL score=0.260	variant of uncertain significance
1 (0)	rs267606661	chr19:45412358	C	G	SNV	missense_	c.805C>G	p.Arg269Gly	0.03605	BS1, PP4 [2], REVEL=0.581	variant of uncertain significance
1 (0)	rs1969839083	chr19:45411157-45411160	CTGT	-	deletion	frameshift	c.184_187del	p.Glu63ArgfsTer15	-	PVS1, PM2, PP4[3]	pathogenic
1 (1)	-	chr19:45411985-45412012	CGGCCAGAG CACCGAGGA GCTGCGGGTG	-	deletion	frameshift	c.432_459del	p.Gly145AlafsTer97	-	PVS1, PM2	likely pathogenic

[1] gnomAD version 2.1.1, the allele frequencies were obtained from exome and genome data, accessed on 16 May 2023.

[2] Three subjects in present study had a clinical data (one male, 48 years old, triglyceride level 3.27 mmol/L, glucose 6.72 mmol/L, body mass index 28.82 kg/m^2, carotid atherosclerosis (number of plaques 3 and maximum stenosis 54.0%), femoral atherosclerosis (number of plaques 3 and maximum stenosis 27.0%), coronary heart disease – no, diabetes mellitus – not known; woman, 51 years

old, triglyceride level 1.70 mmol/L, body mass index 39.85 kg/m^2, carotid and femoral atherosclerosis, coronary heart disease – no; and woman, 53 years old, triglyceride level 1.43 mmol/L, body mass index 28.88 kg/m^2, carotid and femoral atherosclerosis, coronary heart disease – no).

[3]—data of the present study (woman, 57 years old, triglyceride level 4.75 mmol/L, Achilles tendon xanthomas).

AF—allele frequency; ACMG/AMP2015—the American College of Medical Genetics and Genomics/Association for Molecular Pathology; BS1—strong evidence of benign impact; gnomAD—Genome Aggregation Database; HGVSc—Human Genome Variation Society coding sequence name; HGVSp—Human Genome Variation Society protein sequence name; PM (1,2)—moderate evidence of pathogenicity; PP (1,3,4,5)—supporting evidence of pathogenicity; PVS1—very strong evidence of pathogenicity; SNV—single nucleotide variant.

References

1. Feussner, G.; Albanese, M.; Mann, W.A.; Valencia, A.; Schuster, H. Apolipoprotein E2 (Arg-136→ Cys), a variant of apolipoprotein E associated with late-onset dominance of type III hyperlipoproteinaemia. *Eur. J. Clin. Invest.* **1996**, 26, 13–23. doi:10.1046/j.1365-2362.1996.83232.x.
2. van den Maagdenberg, A.M.; Weng, W.; de Bruijn, I.H.; de Knijff, P.; Funke, H.; Smelt, A.H.; Gevers Leuven, J.A.; van't Hooft, F.M.; Assmann, G.; Hofker, M.H.; et al. Characterization of five new mutants in the carboxyl-terminal domain of human apolipoprotein E: no cosegregation with severe hyperlipidemia. *Am. J. Hum. Genet.* **1993**, 52, 937-946.
