# Supplementary material for: Applicability of Diagnostic Criteria and High Prevalence of Familial Dysbetalipoproteinemia in Russia: A Pilot Study
Source: Int J Mol Sci. 2023 Aug 24;24(17):13159. doi: 10.3390/ijms241713159 (PMC10487848; doi:10.3390/ijms241713159)
Supplement: Supplementary file 1 [file ijms-24-13159-s001.zip › Supplementary Materials_S2_revised.pdf]

**Characteristics**	**ESSE-Ivanovo sample (*n* = 1652)[1]**
Men, n (%)	614 (37.2%)
Age, years	49 [39; 57]
Body mass index, kg/m^2	28.0 [24.6; 31.8]
Presence of diabetes, n (%)	82 (5.0%)
Presence of arterial hypertension, n (%)	1,228 (74.3%)
Coronary heart disease, n (%)	73 (4.4%)
Statins, n (%)	93 (5.7%)<br>*n* = 1644[2]
Omega-3 polyunsaturated fatty acids, n (%)	1 (0.1%)<br>*n* = 1644[2]
Total cholesterol, mmol/L	5.46 [4.72; 6.28]
Triglycerides, mmol/L	1.23 [0.87; 1.82]
Low density lipoprotein cholesterol, mmol/L	3.20 [2.41; 4.01]
High density lipoprotein cholesterol, mmol/L	1.38 [1.18; 1.62]

Supplementary Table S2. Characteristics of the ESSE-Ivanovo sample.

[1] Data are presented as percentages or median [Q1; Q3]. [2] Eight subjects had no data on lipid-lowering therapy.
